# The Effect of a Single Intravenous Corticosteroid Administration on Pain after Knee Arthroscopy: A Prospective, Double-Blind, Non-Randomized Controlled Clinical Trial

**DOI:** 10.3390/jcm12010197

**Published:** 2022-12-27

**Authors:** Mohammad Hamdan, Bassem I. Haddad, Ula Isleem, Mohamad Yasin, Mustafa Alrabayah, Hashem Al Hawamdeh, Sharifeh Almasaid, Dayana Jibrin, Mohammad Daas, Saif Aldeen AlRyalat, Mohammad Ali Alshrouf

**Affiliations:** 1 Department of Special Surgery, Division of Orthopedics, School of Medicine, The University of Jordan, Amman 11942, Jordan; 2 Department of Orthopedic Surgery, Icahn School of Medicine at Mount Sinai, New York, NY 10029, USA; 3 Department of Anesthesia, School of Medicine, University of Jordan, Amman 11942, Jordan; 4 Department of Special Surgery, School of Medicine, University of Jordan, Amman 11942, Jordan; 5 Department of Special Surgery, Division of Ophthalmology, School of Medicine, University of Jordan, Amman 11942, Jordan; 6 Jordan University Hospital, The University of Jordan, Amman 11942, Jordan

**Keywords:** knee arthroscopy, knee joint, post-operative pain, dexamethasone, morphine, ambulatory surgical procedures

## Abstract

Background: Knee arthroscopy is a standard surgical procedure that is nowadays widely performed as day-case surgery. The aim of the study was to observe the effect of a single dose of intravenous corticosteroid on pain after undergoing knee arthroscopy for non-bony procedures. Methods: A prospective, double-blind study design was adopted. Patients undergoing knee arthroscopy for non-bony procedures were prospectively assigned into two equal groups: control (those who were not given steroids) and treatment (those who were given eight milligrams of dexamethasone intravenously 15 min prior to the inflation of the tourniquet). The pain was assessed pre-operatively on admission and on the first post-operative day during the morning round in five different movements using a visual analogue scale (VAS). Results: A total of 60 patients were included in the study. There was no significant difference in the pre-and post-operatively scores between both groups. The treatment group experienced a significant reduction in post-operative morphine requirements, with 80% of patients who did not receive dexamethasone requiring post-operative morphine compared to only 53.3% of patients who did (*p* = 0.027). Conclusions: Pre-operative intravenous administration of a single dose of dexamethasone may decrease opioid requirements for adequate pain control after knee arthroscopy.

## 1. Introduction

Knee arthroscopy is one of the most common orthopedic procedures. It is used to treat an array of conditions, particularly the repair or debridement of meniscal tears, removal of loose bodies, synovectomy, ligament reconstruction, and meniscal transplantation [1]. Arthroscopic surgery is minimally invasive and causes less trauma than open surgery, but it can nonetheless result in severe early post-operative pain [2,3,4,5]. This pain is speculated to be due to stimulation of free nerve endings and nociceptors in the synovial tissue, penetration of the anterior synovium, fat pad, and joint capsule [6]. It has been reported that 60% of patients complain of at least moderate pain within 24 h of ambulatory surgery [7]. In another study, it was reported that patients who had undergone knee arthroscopy had longer-lasting persistent pain and a higher percentage of patients took analgesic medication compared to those who had laparoscopy [8]. Furthermore, it was reported by Solheim et al. that the incidence of moderate or severe pain after knee arthroscopy is 60% or less [4].

Knee arthroscopic procedures are performed as day cases in many institutions. Adequate post-operative analgesia is even more crucial for day case procedures and should ideally have a prolonged duration of action with minimal side effects to allow the patient to return to regular activity after discharge. Many substances have been studied for this use, including steroids, opioids, local anesthetics, and combinations of the aforementioned, with some demonstrating clinical benefits and others failing to do so; however, there is no consensus between clinicians and researchers on the efficacy of these combinations or their possible side effect [9,10,11,12,13].

Dexamethasone may reduce post-operative discomfort if added to the treatment strategy. Although dexamethasone does not contribute directly to pain pathways, it does reduce inflammatory markers, which can decrease pain levels and shorten hospital stays [14]. Steroid administration to patients has raised concern about the adverse effects on patients undergoing any procedure, especially the increased risk of infection of surgical wounds. Studies found no side effects from a single dose of dexamethasone [15,16,17,18]. In other studies, even a single dose of dexamethasone was found to increase the risk of postoperative infection or cause a mild increase in blood glucose, particularly at higher doses [19,20,21]. Moreover, lowering the opioids’ requirement will limit the serious opioid-related side effects, including but not limited to somnolence, respiratory depression, constipation, nausea and vomiting, and addiction [22]. Moreover, it has been reported previously that 4 mg of dexamethasone pre-operatively reduced the risk of postoperative nausea and vomiting by 26% [23].

Although knee arthroscopy is an extremely common surgery for a variety of conditions, there is no consensus on how to manage the pain and disability after these procedures with minimal side effects. The purpose of our study is to observe the effects of pre-operative corticosteroid administration on acute post-operative pain in patients after undergoing arthroscopic knee procedures. Taking into consideration that glucocorticoids, including dexamethasone, are sometimes used to relieve severe acute pain following orthopedic surgery, we hypothesized that the administration of a single intravenous dose of dexamethasone prior to surgery would reduce the number of opioids required for adequate pain management following knee arthroscopy.

## 2. Materials and Methods

### 2.1. Study Design and Population

This prospective, double-blinded study involved patients who underwent knee arthroscopy for non-bony procedures from 2019 to 2022. A total of 60 patients were enrolled in this study from a single, 600-bed tertiary care teaching hospital during the duration of the study. Patients were eligible for participation if they were undergoing knee arthroscopy for non-bony or soft tissue procedures, were mobile, could cooperate and understand the visual analog score (VAS) scale, and had no contraindications for receiving the medications which included hypersensitivity to dexamethasone or any corticosteroids. Exclusion criteria were cases of soft tissue procedures that needed tunnel drilling of the bone, such as anterior cruciate ligament and collateral ligament surgery or osteochondral autologous transfer surgery (OATS), because they also needed bone drilling and raised pain and sensitivity levels. The primary objective of this study was to evaluate the effect of a single intravenous corticosteroid dose on pain and analgesia requirements after knee arthroscopy.

Prior to the study’s beginning, the protocol was evaluated and approved by the Jordan University Hospital ethics committee, and the appropriate institutional review board (IRB) approved the study proposal (approval number 67/2018/2494; 27 September 2018). The Code of Ethics of the World Medical Association (Declaration of Helsinki) was followed while conducting the study. All patients participating in this study signed written informed consent.

### 2.2. Data Collection

The data were collected on a pre-formed collection sheet that was developed and created by the authors. The sheet included four major sections: patient demographics, covering the patient’s age, weight, height, and BMI. The second section included the pain scores, which were recorded using two instruments twice by an observer who was blind to the chosen group: once a day before the surgery at the time of admission and once the next morning after surgery at the morning round around 9 a.m. (almost 24 h post-operative). The first tool included a physical movement pain score ranging from 0 to 10, with 0 indicating no pain and 10 being extremely painful. Patients were asked by a senior orthopedic resident (5th year resident) to perform a set of five movements at the bedside that included a straight leg raise while lying supine, knee flexion with the patient lying supine, knee extension and flexion while sitting on the couch, and five steps of walking. We chose these 5 movements based on our experience (the most common movement on the knee that causes pain) and to unify the situations for which the pain score was recorded in order to make the pain comparison more objective. The scores were documented accordingly for each movement. A second tool that included a VAS score, which included faces with various expressions and a 0 to 10 pain scale, with 0 indicating no pain and 10 being extremely painful, was used as an additional pain assessment tool. The third section of the data sheet included operation details, considering that all patients underwent arthroscopy for non-bony procedures under spinal anesthesia and tourniquet control, mainly for soft tissue procedures including diagnostic arthroscopy, meniscectomy, or meniscal repair. The fourth section contained additional details regarding any procedures performed, the need for analgesia or opioids within the last 24 h pre-operatively, intraoperatively, or post-operatively, and the dosage details (available as Supplementary Material).

### 2.3. Anesthetic and Surgical Procedure

Patients underwent standard general anesthetic protocols. The patients were not premedicated. In the operating room, the standard monitors were applied to the patients, including an electrocardiogram, pulse oximetry, capnography, and non-invasive blood pressure, an 18-gauge IV cannula was inserted, an infusion of lactated Ringer’s solution was started, and emergency drugs and precautions were prepared for toxicity and other complications. 

Spinal anesthesia was performed for all patients by giving a total volume of 3 mL: 12.5 mg of 0.5% heavy Bupivacaine (2.5 mL) mixed with 25 µg Fentanyl (0.5 mL) and administered intrathecally using a 25 G pencil-point spinal needle under a complete septic technique. The level and potency of the block were assessed before starting the procedure. No local anesthetic intra-articular injections were performed. For intraoperative increases in heart rate or systolic blood pressure greater than 20% above baseline levels, fentanyl was administered in a dose of 50 μg. In the postanesthesia care unit, patients were evaluated for pain using the VAS and numeric pain score, and if the pain score exceeded 4 out of 10, patients were given 1g of IV paracetamol, and if was more than 6 out of 10, morphine, of 2 mg IV every 10 min, was given until the pain score was less than 4.

The surgical technique involves standard knee arthroscopy with three portals: a medial supra-patellar for normal saline inflow, an antero-lateral (AL) for the arthroscope, and an antero-medial (AM) for instruments.

### 2.4. Treatment Protocol 

Patients were assigned by the operating surgeon into two groups: one group received eight milligrams of dexamethasone intravenously (IV) 15 min before inflation of the tourniquet (treatment group) and the other group did not (control group). For convince purposes, the patients were assigned to each group in order of their presentation to the hospital after being determined to be eligible for the study, with the first 30 eligible patients placed in the control group and the following 30 eligible patients assigned to the treatment group. The process was blinded for both the patients and the data collection team to limit the bias, whereas the operating surgeon, the anesthesiologist, and the statistician were not blinded. In addition, all these procedures were performed by a single professional and expert sports medicine surgeon of the knee according to the same protocol, with a torniquet time and procedure time not exceeding 1 h at maximum, and under the supervision of the same senior anesthesiologist.

Both groups were managed post-operatively using the same pain management protocol, including a regular one-gram acetaminophen IV every eight hours and, as needed, intramuscular (IM) morphine according to the patient’s weight (0.5 milligrams per kilogram and a maximum dose of 5 to 6 g). The frequency of ordering pain medication and the dosages were recorded in the data collection sheet. There were no surgical site infections or wound healing complications in any of the cases after two weeks, when patients presented for removing the sutures, or at least after a three-month follow-up period.

### 2.5. Statistical Analysis

Statistical analysis was performed using SPSS version 23.0 (Chicago, IL, USA), and both the mean ± standard deviation and count (frequency) were used to describe continuous variables (e.g., age) and nominal variables (e.g., gender), respectively. Further, an independent sample t-test was used to analyze the mean difference between each group and continuous scale measurements (i.e., age, biometric measurements, and pain scores), where data were represented using the mean ± standard deviation. Chi-square testing was used to analyze the difference in morphine and acetaminophen use among each group. To compare the pain score and visual analogue scores between both groups, the difference was calculated by subtracting the pre-operative score from the post-operative score. Subsequently, a Mann–Whitney *U* test was performed to compare the visual analogue scale between groups. All the underlying assumptions were met, and a *p* value of 0.05 was adopted as a significant threshold.

## 3. Results

A total of 60 patients were included in this study, with a mean age of 34 ± 11.04 years. Of these patients, 73.3% were men and 26.7% were women. Half of the patients (n = 30) received dexamethasone pre-operatively, whereas the other half did not. There were no significant differences between both groups when comparing the demographic characteristics, as shown in Table 1.

When looking at the pain management effect, a significant difference was found regarding morphine use, where 80% of patients who did not receive dexamethasone required post-operative morphine compared to only 53.3% of patients who did (*p* = 0.027). 

Regarding the use of IV acetaminophen, there was no significant difference (*p* = 0.056). There was no significant difference in the pre- and post-operatively mean score between both groups among the physical movement pain scale scores, as shown in Table 2. Furthermore, there was no significant difference in median scores between each group with regard to VAS scores (Table 3).

## 4. Discussion

Our data showed that a single pre-operative IV dexamethasone injection helped achieve adequate post-operative pain control while significantly decreasing post-operative morphine requirements. The control group achieved comparable post-operative pain control; however, with significantly higher morphine requirements. This is explained by the fact that 80% of the control group requested morphine for pain control, compared to only 53% of the treatment group. There was no significant difference in the dosages of post-operative acetaminophen between the patients who were administered perioperative dexamethasone and those who were not.

Uncontrolled pain is an undesirable effect of orthopedic procedures. This could lead to an increased duration of hospital stay and more prolonged time to start rehabilitation, leading to more post-operative complications and subsequently more readmissions, and a higher cost of care [24,25]. A randomized controlled trial on chronic pain showed that 30% of patients had persistent pain a year after arthroscopic knee procedures, with 10% reporting moderate intensity [3]. This study only looked at people with moderate or severe pain right after the surgery, which is a strong sign of long-term pain after surgery. Solheim et al. reported that 67 % of patients experienced moderate or severe pain within 1 h after arthroscopic knee surgery [4].

Ideally, pain management regimens should be efficacious and have minimal side effects. The use of multimodal pain management has been shown to serve this purpose, particularly in orthopedic surgical cases [26,27]. Dexamethasone is not only a potent steroid with minimal side effects, but also a proven anti-inflammatory and anti-emetic [28]. Studies have shown that a single dose of dexamethasone reduces pain as well as post-operative hospital stays [29]. The results of our study demonstrate the additional benefit of single-dose IV dexamethasone administration for post-operative pain control, as evident in the decreased need for morphine. Even though some studies have shown that dexamethasone may be helpful for post-operative analgesia when combined with other drugs, systemic reviews on dexamethasone injections for analgesia in orthopedic surgery, especially knee surgery procedures, did not draw any conclusions [30,31].

Surgical patients undergoing surgery under general anesthesia were found to benefit from a single IV perioperative dose of dexamethasone, which could lead to reduced post-operative pain, lower need for post-operative opioids, longer time to take their first analgesia dosage, less rescue analgesia, and decreased time spent in the post-anesthesia care unit [32]. Regarding knee arthroscopy, a recent meta-analysis of randomized controlled trials was published, which included four randomized control trials on the use of dexamethasone on pain management for knee arthroscopy; they concluded that the use of dexamethasone could provide additional benefits for pain control in patients with knee arthroscopy [33].

A study that included 78 patients by Moyano et al. found that IV dexamethasone during the intraoperative period had no significant clinical impact on post-operative pain intensity during the first 48 h after arthroscopic knee surgery when given in conjunction with another anti-inflammatory [34]. Nevertheless, it was found that perineural and IV dexamethasone had no difference in pain relief after shoulder arthroscopy [35]. Furthermore, in patients having elective knee arthroscopy, it was found that combining dexamethasone or dexmedetomidine with intraarticular bupivacaine improved the quality and duration of post-operative analgesia and reduced the intake of post-operative analgesics [36,37]. Moreover, the combination of dexamethasone with intra-articular morphine and bupivacaine after knee arthroplasty prolongs the duration of analgesia, lowers pain scores, and reduces the total amount if analgesic needed, with no apparent side effects [38]. 

There are some limitations to this study. First, the relatively small sample size reduces statistical power and makes it difficult to identify differences between the groups. Because there were no differences between the two groups’ demographic, the likelihood of a type I error was low. Second, this study may have been affected by regional ethnic sample biases because all patients were recruited from a single center. Third, it should be mentioned that it is important to keep in mind the possibility that even a single dose of dexamethasone may cause side effects. However, our study had several strengths. This includes the prospective nature of the study and the specific movements for which pain scores were recorded, which makes them more objective. We used a double-blind design to ensure that neither the experimenter nor the participants could be biased. Furthermore, all of the operations were performed by the same experienced orthopedic surgeon using the same protocol to avoid any element of variation across patients. Therefore, we believe that our findings provide preliminary evidence that offers a rationale for future clinical studies with a larger sample size. Moreover, we recommend future researchers to also study the effect of dexamethasone on postoperative nausea and vomiting, which could be an additional advantage of using it, as it was previously reported that 4 mg of dexamethasone given pre-operatively reduced significantly the risk of postoperative nausea and vomiting [23].

## 5. Conclusions

The results of this study showed that pre-operative administration of a single dose of intravenous dexamethasone decreased the need for opioids required for adequate post-operative pain control. Further studies on larger samples may be required.

## Figures and Tables

**Table 1:** Characteristics of the treatment and control groups.

	Treatment Group (n = 30)		Control Group (n = 30)		*p* Value
	Count (%)	Mean ± SD	Count (%)	Mean ± SD	
Gender					
Male	24 (80.0)		20 (66.7)		0.191
Female	6 (20.0)		10 (33.3)		
Age (years)		35.00 ± 11.41		33.00 ± 10.76	0.488
Weight (kg)		81.23 ± 19.09		77.07 ± 15.16	0.353
Height (cm)		171.67 ± 8.13		167.90 ± 9.55	0.105
BMI (kg/m^2^)		27.35 ± 4.88		27.20 ± 4.03	0.899

SD, standard deviation; BMI, body mass index; n, *p*.

**Table 2:** Pre-operative (pre-op) and post-operative (post-op) mean ± standard deviation pain scores during five tasks with their mean difference (post-op minus pre-op scores) for both groups.

Pain Score	Treatment Group		Control Group		*p* Value
	Mean ± SD	Score Difference between Post-Op and Pre-Op ± SD	Mean ± SD	Score Difference between Post-Op and Pre-Op ± SD	
Straight leg raising test pre-op	2.40 ± 3.01	2.30 ± 3.60	2.17 ± 2.78	1.63 ± 3.32	0.459
Straight leg raising test post-op	4.70 ± 3.58		3.80 ± 3.66		
Knee flexion supine pre-op	4.03 ± 3.31	1.97 ± 3.10	3.10 ± 3.09	1.93 ± 2.55	0.964
Knee flexion supine post-op	6.00 ± 2.57		5.03 ± 2.85		
Knee extension pre-op	3.07 ± 2.92	2.00 ± 3.54	3.33 ± 3.28	1.47 ± 3.26	0.546
Knee extension post-op	5.07 ± 3.16		4.80 ± 2.63		
Knee flexion sitting pre-op	2.87 ± 3.20	2.57 ± 3.04	2.80 ± 2.95	2.90 ± 3.96	0.333
Knee flexion sitting post-op	5.43 ± 2.92		5.70 ± 3.19		
Five steps walking pre-op	3.53 ± 3.58	2.37 ± 3.70	3.23 ± 3.52	1.60 ± 3.77	0.43
Five steps walking post-op	5.90 ± 3.14		4.83 ± 3.38		

SD, standard deviation; the *p* value represents the difference between both groups with regards to the mean pre-and postoperative score difference; all *p* values were calculated using independent sample t-test.

**Table 3:** Pre-operative (pre-op) and post-operative (post-op) median (first to third percentiles) visual analogue scale during five tasks with their median difference (post-op minus pre-op) for both groups.

Visual Analogue Scale	Control Group		Treatment Group		*p* Value
	Median (1st to 3rd Percentiles)	Score Difference between Post-Op and Pre-Op	Median (1st to 3rd Percentiles)	Score Difference between Post-Op and Pre-Op	
Straight leg raising test pre-op	2 (1–3)	0 (0–1)	1 (1–3)	1 (0–2)	0.796
Straight leg raising test post-op	2 (1–4)		3 (1–5)		
Knee flexion supine pre-op	2 (1–4)	0.5 (0–2)	3 (1–4)	1 (0–2)	0.789
Knee flexion supine post-op	3 (3–5)		4 (3–4)		
Knee extension pre-op	2.5 (1–4)	0.5 (−1–2)	2 (1–3)	1 (0–2)	0.784
Knee extension post-op	4 (3–4)		3 (2–4)		
Knee flexion sitting pre-op	2 (1–4)	0.5 (0–3)	2 (1–4)	1 (0–2)	0.794
Knee flexion sitting post-op	4 (2–5)		3 (3–4)		
Five steps walking pre-op	2 (1–4)	1 (0–2)	2 (1–5)	0 (0–2)	0.796
Five steps walking post-op	4 (2–5)		4 (2–5)		

The *p* value represents the difference between both groups with regards to median post-op-pre-op score difference; all *p* values were calculated using Mann–Whitney *U* test.

## Data Availability

The raw data from the present research that were utilized and analyzed are available online as a supplementary material.

## Supplementary Materials

The following supporting information can be downloaded at: https://www.mdpi.com/article/10.3390/jcm12010197/s1, The raw data from the present research that were utilized and analyzed are available online as a supplementary material.
